# Molecular and Pharmacokinetic Aspects of the Acetylcholinesterase-Inhibitory Potential of the Oleanane-Type Triterpenes and Their Glycosides

**DOI:** 10.3390/biom13091357

**Published:** 2023-09-06

**Authors:** Katarzyna Stępnik, Wirginia Kukula-Koch, Wojciech Płaziński

**Affiliations:** 1 Department of Physical Chemistry, Institute of Chemical Sciences, Faculty of Chemistry, Maria Curie-Sklodowska University in Lublin, Pl. M. Curie-Skłodowskiej 3, 20-031 Lublin, Poland; 2 Department of Pharmacognosy with Medicinal Plants Garden, Medical University of Lublin, ul. Chodźki 1, 20-093 Lublin, Poland; virginia.kukula@gmail.com; 3 Department of Biopharmacy, Medical University of Lublin, ul. Chodźki 4a, 20-093 Lublin, Poland; wojtek_plazinski@o2.pl; 4 Jerzy Haber Institute of Catalysis and Surface Chemistry, Polish Academy of Sciences, ul. Niezapominajek 8, 30-239 Kraków, Poland

**Keywords:** acetylcholinesterase inhibitory test, molecular docking, blood–brain barrier permeation, memory impairment, AChE inhibition kinetics

## Abstract

The acetylcholinesterase-inhibitory potential of the oleanane-type triterpenes and their glycosides from thebark of *Terminalia arjuna* (Combreatceae), i.e.,arjunic acid, arjunolic acid, arjungenin, arjunglucoside I, sericic acid and arjunetin, is presented. The studies are based on in silico pharmacokinetic and biomimetic studies, acetylcholinesterase (AChE)-inhibitory activity tests and molecular-docking research. Based on the calculated pharmacokinetic parameters, arjunetin and arjunglucoside I are indicated as able to cross the blood–brain barrier. The compounds of interest exhibit a marked acetylcholinesterase inhibitory potential, which was tested in the TLC bioautography test. The longest time to reach brain equilibrium is observed for both the arjunic and arjunolic acids and the shortest one for arjunetin. All of the compounds exhibit a high and relatively similar magnitude of binding energies, varying from ca. −15 to −13 kcal/mol. The superposition of the most favorable positions of all ligands interacting with AChE is analyzed. The correlation between the experimentally determined IC_50_ values and the steric parameters of the molecules is investigated. The inhibition of the enzyme by the analyzed compounds shows their potential to be used as cognition-enhancing agents. For the most potent compound (arjunglucoside I; ARG), the kinetics of AChE inhibition were tested. The Michaelis–Menten constant (Km) for the hydrolysis of the acetylthiocholine iodide substrate was calculated to be 0.011 mM.

## 1. Introduction

Neurodegenerative diseases with memory impairment constitute a growing health issue in aging populations worldwide. Life extension in the last century resulting from the progress in medicine is related to the increasing neurodegenerative disease morbidity, especially in developed countries. The World Health Organization has recognized dementia as a public health priority. In 2017, the World Health Assembly endorsed the “Global action plan on the public health response to dementia 2017–2025” [1]. According to the WHO report, in 2015, dementia affected 47 million people worldwide (or roughly 5% of the world’s elderly population). This number is predicted to increase to 75 million in 2030 and 132 million by 2050 [1]. Therefore, one of the key research requirements is to increase the effectiveness of existing therapeutic strategies and the development of new therapeutic strategies as regards the treatment of neurodegenerative diseases with memory impairment [2].

Acetylcholinesterase (AChE) is an enzyme excreted to the synaptic cleft during the formation of the action potential of a neuron in the brain. To memorize or resume memories, the chemical activity of neurons is sustained by the excretion of acetylcholine (ACh) to the synaptic cleft. ACh is then targeting the post-synaptic membrane where it binds with cholinergic receptors to sustain the action potential. Later, an excessive amount of ACh is decomposed by the enzyme, namely AChE, to prepare the neuron for the following activities. However, in the elderly, a decreased volume of ACh is excreted to the synaptic cleft together with an elevated volume of AChE enzyme, which, in fact may lead to a significant reduction in the action potential number or strength [3]. The described changes on the chemical level in the brain trigger memory impairment effects. Thus, the inhibition of the AChE enzyme was found to stimulate neuronal functions and by this action led to improved cognition. The inhibitors of the AChE enzyme are now the first-line drugs in the treatment of dementia and Alzheimer’s disease (AD) [4,5,6]. Even if there are several registered drugs from the group of AChE inhibitors on the market, there is still a need for new, more tolerable, longer-acting and stronger compounds. 

Due to their numerous health-promoting properties, natural products play a very important role in drug discovery. Initially, the possibility of their use was sought mainly in the treatment of cancer, infectious diseases and cardiovascular diseases [7]. Currently, natural products are of interest to many researchers around the world. Plant-derived secondary metabolites have been widely studied for numerous pharmacological properties, including the treatment of dementia and diseases related to memory disorders. Plant-derived secondary metabolites including oleanane-type triterpenes and their glycosides have provento be interesting sources of compounds with neuroprotective, as well as memory-enhancing, effects, e.g., medicagosides A-F from *Medicago sativa* L. [8,9], onjisaponins isolated from the roots of *Polygala tenuifolia* [10,11] or platycodins from *Platycodi radix* [12]. Also, the *Terminalia arjuna* (Combreatceae), being a valued tree widely used in Indian traditional medicine, seems to be worthy of interest as a natural source of compounds with a cognition-enhancing potential, e.g., arjunolic acid with a neuron-protective potential againstoxidative-stress-associated damage [13].

The study described herein will focus on the determination of AChE-inhibitory potential by naturally occurring oleanane-type triterpenes and their glycosides, as well as the elucidation of the structural pattern of the interactions between the studied compounds and the AChE enzyme employing the ligand–protein docking methodology. The recognition of the AChE-inhibitory potential will be performed using the thin layer chromatography (TLC) bioautography method. In addition, both values of pharmacokinetic and physicochemical parameters connected with the blood–brain barrier (BBB) permeation will be determined using computational and biomimetic methods. These are used as screening tests in the drug discovery process [14] to study the broad spectrum of biological activity, including the ability to cross specific biological barriers. The BBB is a selective barrier, with the endothelium forming a much tighter interface than peripheral endothelia because the gaps between the capillary endothelial cells in most parts of the brain are sealed by tight junctions and thus have a severely limited permeability [15]. The experimental determination of BBB permeability based on in vivo studies requires complex techniques which are usually time-consuming and expensive [14,16]. Mainly due to ethical and economic reasons, in vivo experiments should be preceded by alternative tests including computational and biomimetic ones [17]. This is in line with Green Chemistry principles [18] and with the European Union Directive (Directive 2010/63/EU) which is based on the Three Rs principle of replace, reduce and refine regarding the exploitation of animals for scientific purposes. 

## 2. Materials and Methods

### 2.1. The Analytes

The chemical structures of the investigated oleanane-type triterpenes and their glycosides of plant origin are presented in Table 1.

### 2.2. Chemicals

The pharmacopeial standards of arjunic acid, arjunolic acid, arjungenin, arjunglucoside I, sericic acid and arjunetin were purchased from Sigma Aldrich (St. Louis, MO, USA; p.a.). The organic modifiers for the micellar mobile phase, i.e., acetonitrile and isopropanol, as well as surfactants: polyoxyethylene (23) lauryl ether (Brij35) and dodecyl sodium sulfate (SDS), were purchased from Merck (Darmstadt, Germany; p.a.). The buffer components, i.e., citric acid and disodium hydrogen phosphate (Na_2_HPO_4_), were purchased from Sigma Aldrich (Sigma Aldrich, St. Louis, MO, USA; p.a.). Distilled water was obtained from the Direct-Q3 UV apparatus (Millipore, Warsaw, Poland).

### 2.3. Chromatographic Equipment

At the stage of biomimetic research, high-performance liquid chromatography (HPLC) was used. The Shimadzu Vp liquid chromatographic system (Shimadzu, Kyoto, Japan) equipped with an LC 10AT pump, SPD 10A UV-Vis detector, SCL 10A system controller, CTO-10 AS chromatographic oven and Rheodyne injector valve with a 20 µL loop was applied in the HPLC measurements. 

### 2.4. Chromatographic Conditions

The solutions of pharmacopeial standards of the studied compounds were prepared in methanol (Merck, Darmstadt, Germany; p.a.) at a concentration of 1 mg/mL. All of the oleanane-type triterpenes and their glycosides proved to be in the neutral form in solution under experimental conditions. The optimization process of the chromatographic separation was performed before the experiment. The flow rate of the mobile phases was established to 1 mL/min and the temperature was set at 25 °C. The tested compounds were detected with the UV light at 210 nm. 

The C18 encapped column (Purosphere; 125 × 4 mm i.d., 5 µm; Merck, Darmstadt, Germany) was used as the stationary phase, while the buffered solutions of both Brij35 and SDS were used as the mobile phases. The mobile phases’ compositions were as follows: Brij35 at the concentrations: 0.04; 0.06; 0.08; 0.10 mol/dm^3^ (pH 7.4) with the addition of acetonitrile (10% *v*/*v*); SDS at the concentrations: 0.06; 0.08; 0.10; 0.12 mol/dm^3^ (pH 7.4) with an addition of isopropanol as an organic modifier (7% *v*/*v*). The buffer was prepared from the solutions of both Na_2_HPO_4_ (0.02 mol/dm^3^) and citric acid (0.01 mol/dm^3^). 

The dead time values were measured from the citric acid peaks. All of the reported logarithms of the retention factor were measured three times. The values of peak asymmetry factor were in the acceptable range. 

### 2.5. Pharmacokinetic In Silico Studies

All of the BBB-pharmacokinetic descriptors were calculated using the ACD/Percepta software (version 2012, Advanced Chemistry Development, Inc., Toronto, ON, Canada).

### 2.6. TLC-Based Bioatographic Assay towards the AChE-Inhibitory Activity

Six standards of oleanane-type triterpenes: arjunic acid, arjunolic acid, arjungenin, arjunglucoside I, sericic acid and arjunetin—purchased from Sigma Aldrich (St. Louis, MO, USA)—were prepared at the concentration of 1 mg/mL in double-distilled water: methanol (50:50 *v*/*v*) and they were applied separately at the surface of the aluminum normal phase 10 cm × 10 cm TLC plate (Silica gel 60 F254, Merck, Darmstadt, Germany) with an autosampler (Camag, Muttenz, Switzerland) as the 6 mm zones, distant from one another by 1.5 cm horizontally and 2 cm vertically. Every reference solution was applied as 4, 6, 8 and 10 µL volume bands on three TLC plates.

The TLC plates were later subjected to the enzymatic assay according to the previously published protocol [19], with some modifications.

As the TLC plate was not developed in a TLC solvent system but was used directly in the TLC bioautographic assay, the authors modified the previously published protocol and sprayed the TLC with the substrate (2-naphtyl acetate; Sigma Aldrich, St. Louis, CA, USA) dissolved in distilled water at the quantity of 30 mg/20 mL. The TLC was dried in cold air and later the solution of the AChE enzyme (AChE from electric eel type VI-S, Sigma Aldrich, St. Louis, CA, USA) dissolved in the aqueous solution of Tris-HCl buffer (pH 7.8; Sigma Aldrich, St. Louis, CA, USA) with bovine serum (500 mg/100 mL, Sigma Aldrich, St. Louis, CA, USA) at the quantity of 3 U/mL was sprayed on the TLC plate and incubated at the temperature of 37 °C for the following 20 min in a humid incubator. In the next step, the Fast Blue B (Sigma Aldrich, St. Louis, CA, USA) solution (0.615 mg/mL) was sprayed on the plate and visualized active zones as white spots against the violet background. The area of the discolored zones corresponded to the inhibitory strength of the respective zones.

Finally, the TLC plate was dried in the air and analyzed by a Camag TLC visualizer (Camag, Muttenz, Switzerland) under visible light. The peak areas of the discolored zones were automatically calculated by the WinCats program (version 1.4, Camag, Muttenz, Switzerland) and their size was compared to calculate the IC_50_ values that corresponded to the concentration of the standard giving the half maximum inhibition of the AChE enzyme. 

### 2.7. Molecular-Docking Procedure

The ligand molecules were obtained by the online SMILES translator (cactus.nci.nih.gov/translate) and subsequently optimized by using Avogadro 1.1.1 [20] and the UFF force field [21] (5000 steps, steepest descent algorithm). Flexible and optimized ligand molecules were docked into the binding pocket of the protein structure found in the PDB database (PDB:1EVE). Docking simulations were carried out in the AutoDockVina software (version 1.1.2) [22]. The procedure was performed within the cuboid region of dimensions of 22 × 30 × 34Å^3^ which covers the co-crystallized ligand present in the considered PDB record, as well as the closest amino-acid residues that exhibit contact with this ligand. All of the default procedures and algorithms implemented in AutoDockVina were applied during the docking procedure. The rotatable torsional angles in both ligand molecules and the selected amino-acid sidechains within the binding cavity (Tyr334, Phe330, Phe75, Trp84, Glu199, Ser200, Tyr70, Tyr121, Trp279, Phe290, Phe331, Phe288, His440, Gln74, Leu282, Trp432, Asn85 and Asp285) were allowed to rotate. Visual inspections of each pose of the docked ligands were carried out in order to assure that the binding energies correspond to the structurally analogous orientations. The procedure was validated in our previous work [23].

### 2.8. Kinetics of AChE Inhibition

The samples of the most potent AChE inhibitor, i.e., ARG, were prepared in 12 dilutions in the concentration range of 0.00045–0.92 mM in dimethyl sulfoxide (DMSO ≥ 99.7%; Sigma Aldrich, St. Louis, CA, USA). Ellman’s colorimetric method [24] was applied with some modifications [25]. Each of the tested ARG samples (15 μL) was mixed with 20 μL of the AChE solution (from electric eel, Type VI-S; Sigma Aldrich, St. Louis, CA, USA; 0.28 U/mL) and completed after 5 min with 35 μL of acetylthiocholine iodide (ATChI; Sigma Aldrich, St. Louis, CA, USA; 1.5 mmol/L), 175 μL of 0.3 mmol/L 5,5′-dithiobis-2-nitrobenzoic acid (DTNB, containing 10 mmol/L NaCl and 2 mmol/L MgCl_2_; Sigma Aldrich, St. Louis, CA, USA) and 100 μL of Tris-HCl buffer (Sigma Aldrich, St. Louis, CA, USA 50 mmol/L, pH 8.0). The AChE, ATChI and DTNB solutions were prepared in the Tris-HCl buffer (Sigma Aldrich, St. Louis, CA, USA). In order to eliminate the absorbance increase due to the spontaneous hydrolysis of the substrate, “blank” samples were used composed of 15 μL of Tris-HCl buffer instead of ARG, as well as undergoing the above-mentioned compounds. The absorbance of the test samples was measured every minute for 32 min and it was subtracted from the absorbance of the “blank” sample. The background samples were prepared with 15 μL of each ARG solution and 330 μL of Tris-HCl buffer. The samples were incubated at room temperature for 30 min. The absorbance was measured at 412 nm (96-well microplate reader, Tecan Sunrise, Grödig, Austria). Each sample was analyzed in three repetitions. The linear regression analysis was conducted using the Minitab Statistical Software (version 18.1, Minitab Inc., State College, PA, USA) and the values of the correlation coefficients, slopes, intercepts and the standard errors were obtained. 

### 2.9. Toxicity Assay

To assess the ARG toxic effect, the ECOSAR (version 1.11) free software was employed. Based on the ARG chemical structure, both the acute and chronic toxicity endpoints for fish, aquatic invertebrates (Daphnia) and green algae were measured. 

## 3. Results

### 3.1. The BBB-Pharmacokinetic In Silico Studies

The BBB pharmacokinetic descriptors were determined in silicousing the ACD/Percepta software. The following parameters were calculated: logBB—the distribution of a substance in the blood–brain area (the BBB penetration descriptor), logPS—the rate of passive diffusion/permeability (the permeability–surface area product), logPS,_Fu,brain_—the brain/plasma equilibration rate, Fu—the fraction unbound in plasma, and Fb—the fraction unbound in brain (Table 2).

### 3.2. The BBB-Biomimetic Studies

To determine the BBB permeability of the tested oleanane-type triterpenes and their glycosides, micellar chromatographic systems recognized as biomimetic were applied. For this purpose, both the method of biopartitioning micellar chromatography (BMC), using the non-ionic surfactant polyoxyethylene (23) lauryl ether (Brij35), and SDS–micellar chromatography using the anionic dodecyl sodium sulfate (SDS) were applied. 

The relationship between the surfactant concentration in the effluent and the retention of analytes is described by Foley’s equation [26]:

1/*k* = (*K_MA_*/km)*C_M_* + 1/km (1)

where *k* is the retention factor, *C_M_* is the total surfactant concentration in the mobile phase minus the critical micellization concentration (CMC), *K_MA_* is the analyte–micelle association constant, and km is the micellar retention factor at zero micelles concentration in the mobile phase which corresponds to the monomer surfactant concentration equal to CMC. These parameters describe in the simplest way possible the interactions in the micellar system that mimic the biological environment. In this case, both Brij35 and SDS micelles can be treated like a simple BBB model. 

**Figure 1:**
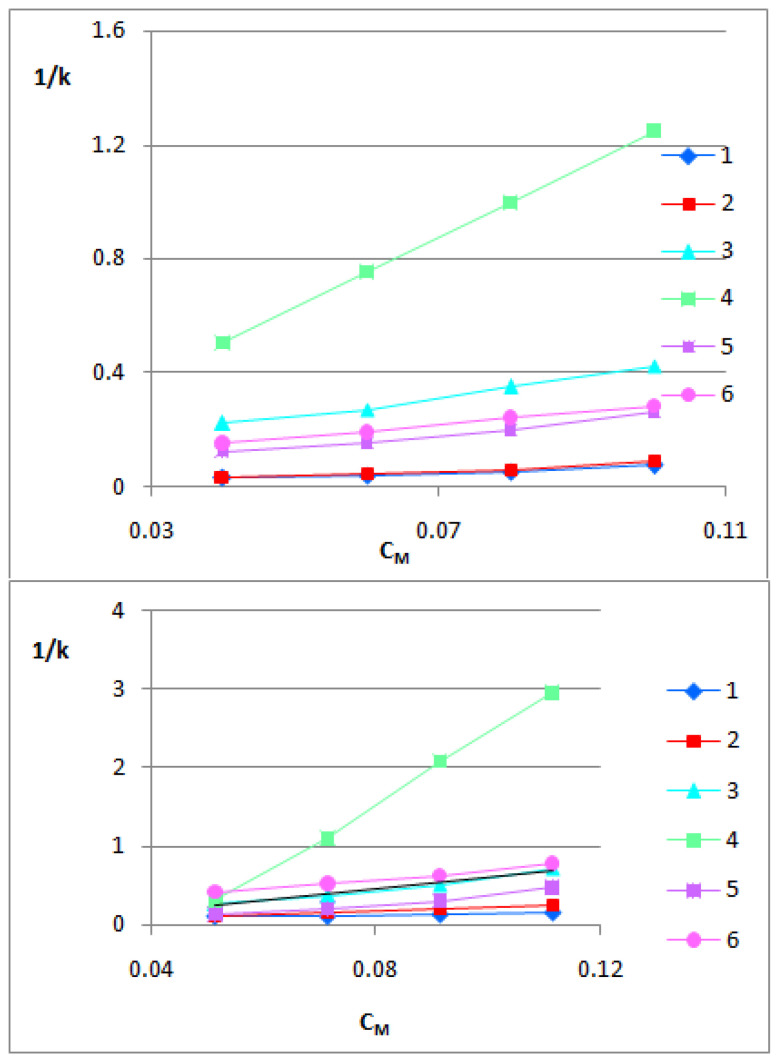
The 1/*k* vs. *C_M_* relationships obtained for the tested triterpenes: arjunic acid (1), arjunolic acid (2), arjungenin (3), arjunglucoside I (4), sericic acid (5) and arjunetin (6) usingthe BMC (**above**) and SDS (**below**) systems.

To evaluate the *K_MA_* and km values, the relationship between the experimental 1/*k* and *C_M_* values should be plotted and then calculated from the slope and intercept of the plot. Very good linear relationships were obtained with the average values of R^2^ equal to 0.96 for BMC and 0.99 for the SDS system. In Figure 1, 1/*k* vs. *C_M_* relationships are presented.

**Figure 2:**
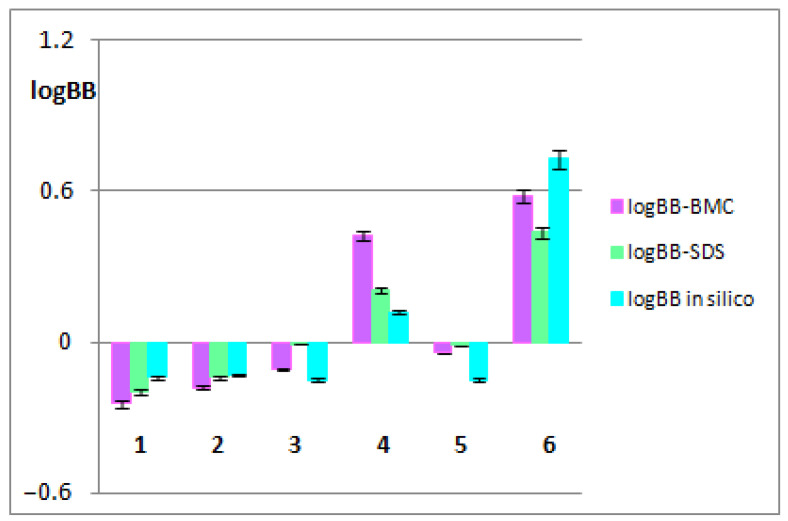
LogBB values obtained for the tested triterpenes: arjunicacid (1), arjunolic acid (2), arjungenin (3), arjunglucoside I (4), sericic acid (5) and arjunetin (6) using biomimetic and computational methods.

Based on Foley’s model, both log *K_MA_* and log km were calculated. These parameters are considered as lipophilicity descriptors due to the affinity to the surfactant-modified stationary phase (km), as well as binding to the micelles (*K_MA_*) [27]. The logarithm of the micellar retention factor, log km, is analogous to the logarithm of the retention factor extrapolated to pure water (log kw) obtained in an RP-LC system with the water–organic mobile phase. In this study, the log (km/*K_MA_*) values calculated from the slopes of Equation(1) were taken as the micellar lipophilicity descriptors, whereas the logarithm of the analyte–micelle association constant (log*K_MA_*) values obtained from micellar systems can be taken as an estimate of logBB values. The calculated logBB values based on the log*K_MA_* obtained from both the BMC and SDS systems (logBB-BMC and logBB-SDS, respectively), as well as logBB in silico values, are presented in Figure 2.

### 3.3. AChE-Inhibitory Activity of the Selected Saponins in the TLC-Bioautography Assay

**Figure 3:**
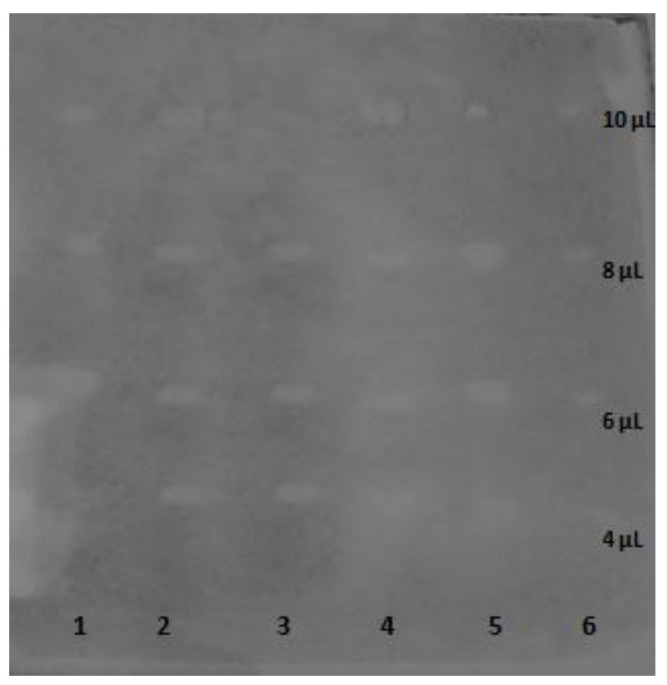
Result of the TLC-bioautography assay for the inhibition of the acetylcholinesterase enzyme on the silica gel showing different concentrations of the tested triterpenes: arjunic acid (1), arjunolic acid (2), arjungenin (3), arjunglucoside I (4), sericic acid (5) and arjunetin (6).

The selected assay is used to search for the AChE-inhibitory properties of single components or ingredients of mixtures that were introduced on a TLC plate. The TLC-bioautography assay was performed on a series of four dilutions of six reference solutions of arjunic acid, arjunolic acid, arjungenin, arjunglucoside I, sericic acid and arjunetinand provided evidence for the AChE-inhibitory properties of all selected triterpenes (Figure 3) that were dependent on the introduced concentration. As presented in Figure 3, the compounds were characterized by a similar inhibitory potential, in similarapplication volumes.

The imaging program (WinCats, Camag) enabled a relative quantitative analysis of the inhibition zones. As a result, from the zones of inhibition, peak areas were obtained. The transformation was necessary to calculate the IC_50_ values of every tested compound to compare their inhibitory potential towards the AChE enzyme (Table 3).

### 3.4. Molecular-Docking Studies

**Figure 4:**
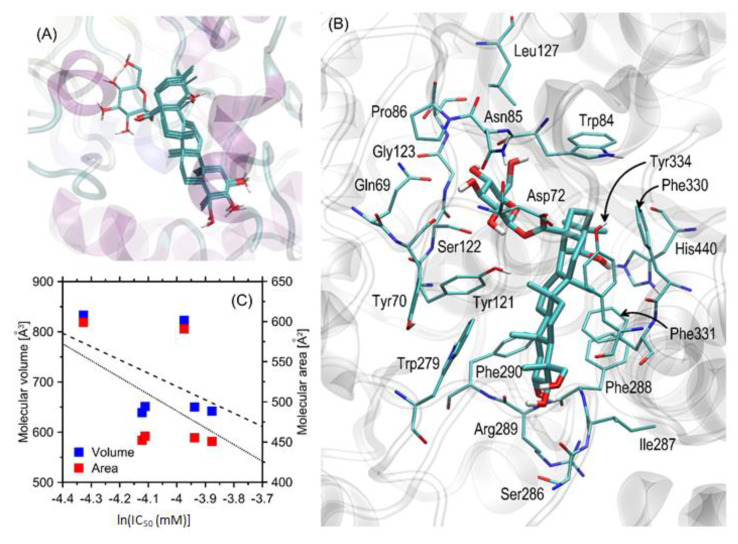
(**A**) The superposition of the most favorable poses of all ligands interacting with AChE. (**B**) The most favorable location of the arjunglucoside I molecule bound to AChE. The ligand molecule is shown as thick sticks, whereas all of the closest amino-acid residues (of a distance no longerthan 0.4 nm) are represented by thin sticks. The description of the interaction types is given in the text. The residue numbering is compatible with the PDB:3EVE record. (**C**) Linear correlations between the experimentally determined IC_50_ values (recalculated as ln(IC_50_)) and the molecular volume (blue points) or molecular area (red points) of the studied compounds, represented by the dashed or solid line, respectively.

All of the compounds exhibit a high and relatively similar magnitude of binding energies, varying from ca. −15 to −13 kcal/mol. All energy values are negative which clearly speaks for strongly favorable binding in all considered cases. Contrary to our previous results, we did not observe any statistically significant correlation between either the experimentally determined IC_50_ values or the theoretically predicted binding energies and molecular dimensions of the studied molecules (i.e., molecular volume and molecular surface area determined by using the 3vee.molmovdb.org online server (accessed on 5 June 2023) with a probe of 0.1 nm and a high grid resolution; Figure 4C). Such an observation suggests that the intensity of binding to AChE is governed by those fragments of molecules which are common for all considered compounds. This is in line with the experimentally observed small scatter of the IC_50_ values. 

### 3.5. The Kinetics of AChE Inhibition

**Figure 5:**
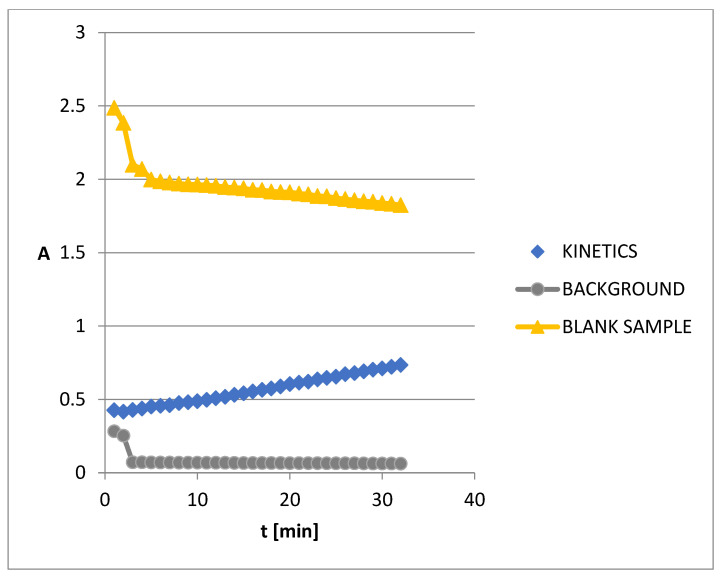
Absorbance (A) vs. time (min) for 0.00045 mM of ARG.

**Figure 6:**
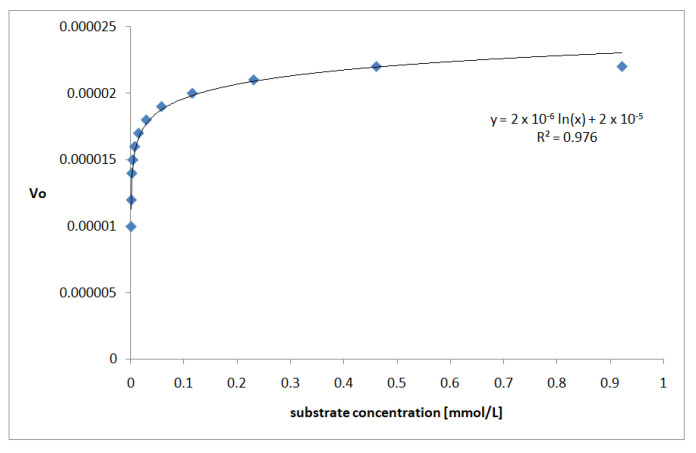
Lineweaver–Burk plot for initial velocity (Vo) vs. substrate concentration (mmol/L).

The absorbance (A) vs. time (min) relationships were plotted (Figure 5) for each ARG concentration. The average value of the correlation coefficient was found to be 0.991. Due to the great linearity, further kinetic studies were carried out. The following basic kinetic enzyme parameters were calculated: the Michaelis-Menten constant (Km) by means of the Lineweaver–Burk plot (Figure 6) and the maximum reaction velocity (Vmax). 

### 3.6. In Silico Prediction of Acute and Chronic Toxicity

Acute toxicity (short-term exposure) was assessed for ARG using the lethal or effect concentration 50 (LC50 and EC50, respectively), whereas chronic toxicity (long-term exposure) was assessed using chronic values (ChV) obtained for fish, Daphnia and green algae (Table 4).

## 4. Discussion

Natural products of plant origin can be interesting sources of compounds with neuroprotective properties. Among them, traditional Chinese medicine (TCM) herbs play an important role, e.g., *Ginkgo biloba* L. [28], *Panax ginseng* [29,30,31,32,33] or *Scutellariabaicalensis* [34,35,36,37,38]. Other plants are also crucial sources of a variety of compounds acting on the central nervous system (CNS), e.g., *Oleaeuropaea* L. [38,39,40], *Vitisvinifera* L. [38,39,40], *Salvia officinalis* L. [41,42,43,44,45], *Melissa parviflora* [46], *Berberisintegerrima* [47] or *Carissa edulis* [48]. The most important chemical groups of such compounds are saponins [49,50], tannins [51,52], flavonoids [53,54], alkaloids [55], etc. Among the above-mentioned groups of the CNS-active compounds are triterpenes and their glycosides [56,57,58,59]. These compounds can affect the CNS including the nerve cells of the brain and spinal cord which control many direct body functions and behavior. In the context of neuroprotective properties, firstly it is important to confirm the ability of a compound to cross the BBB.

*Terminalia arjuna* accumulates bioactive triterpene glycosides (saponins) and aglycones (sapogenins) in a tissue-preferential manner [60]. Many triterpenes demonstrate therapeutic efficacy. In most cases, they can cross the BBB and may affect the CNS including the nerve cells of the brain and spinal cord which control many direct body functions and behaviors. They may also affect the autonomic nervous system which includes the regulation of internal organs, heartbeat, circulation and breathing.

Oleanane triterpenoids/saponins (derived from β-amyrin) have also been reported to have a mainly cardioprotective potential [60,61,62,63]. Moreover, numerous studies have confirmed their antioxidant [64], antimicrobial [65,66], anti-inflammatory [67], anticancer [68], precognitive [13] and hepatoprotective [69] activities, among others. 

It should be strongly emphasized that only the drug fraction unbound in media such as plasma can be transferred into body tissues. Certain in vitro methods including ultrafiltration or equilibrium dialysis are most often used to measure the fraction unbound value of a drug. These in vitro obtained values are used not only for measurement of the transfer rate into body tissues but also of the BBB permeability [70]. It should be remembered that research on the penetration of compounds through the biological barriers, including the BBB one, is carried out using in vivo methods in particular. However, for ethical and economic reasons, the need to use alternative methods other than in vivo ones, including the non-cell based-in vitro (biomimetic) and/or in silico (computational), has been emphasized in recent years. 

Both biomimetic and computational BBB-pharmacokinetic studies are commonly used in laboratory practice at the first stages of an experiment on biologically active compounds (potential drugs) and constitute an important stage of research in the drug design process. At the stage of in silico studies the most important BBB-pharmacokinetic descriptors are calculated, i.e., the distribution of a substance in the blood–brain area, the rate of passive diffusion/permeability, the brain/plasma equilibration rate, the fraction unbound in plasma and the fraction unbound in the brain. The blood–brain distribution (BB), frequently expressed as logBB, is defined as a ratio between the concentration in the brain and the concentration in the blood [71,72]. This experiment first identified two out of six tested compounds, i.e.,arjunetin and arjunglucoside I, capable of crossing the BBB. However, it is commonly recognized that the most important parameter in permeability through the BBB is the permeability–surface area product (PS) often expressed as logPS. These index is closely related to the cerebral blood flow (CBF) which is measured using various invasive, as well as non-invasive, techniques, i.e., direct intravascular measurements, nuclear medicine, X-ray imaging, magnetic resonance imaging, ultrasound techniques, thermal diffusion and optical methods. The most invasive methods require surgical access, arterial puncture or catheterization, while less invasive methods demand the intravenous injection of a contrast agent [73]. The CBF is a very important parameter for brain viability and its functions because it ensures the proper delivery of oxygen which is necessary for the neuronal oxidative metabolism of energy substrates. It is defined as the blood volume that flows per unit mass per unit time in brain tissue and is typically expressed in units of mL blood/(100 g_tissue_ × min), or mL blood (100 mL_tissue_ × min) [73] or in mL blood/(h × kg) [74]. Taking into account the PS values calculated in this experiment, arjunetin exhibited the highest BBB-permeability potential, followed by arjungenin, arjunglucoside I and sericic acid (ex aequo), whereas both acids: arjunic and arjunolicacidexhibited the lowest BBB permeability. 

The scientific reports indicate that the time to reach brain equilibrium can be prolonged when the BBB permeability–surface area product (PS) or the fraction unbound in the brain decreases [75]; therefore, it can be noticed that the lower the values of the PS or Fb, the longer the time required to reach brain equilibrium [74]. In our experiment, no significant differences between Fb values were observed, whereas the differences between the PS values were much greater (from 0.63 mL × h^−1^ × kg^−1^ for both arjunic and arjunolic acids to 5 mL × h^−1^ × kg^−1^ for arjunetin). Then, the longest time to reach brain equilibrium can be observed for the above-mentioned acids and the shortest for arjunetin. A high rate of penetration results from high BBB permeability, as well as low brain tissue binding [74].

In addition, analyzing values from Table 2, it can also be seen that arjunetin and arjunglucoside I bind the least to blood plasma proteins (the highest value of free drug concentration, Fu) and these compounds show the highest log BB values (0.73 and 0.12, respectively). The rest of the compounds have logBB values less than zero. Therefore, it can be presumed that among the tested compounds, arjunetin and arjunglucoside I are the substances that can penetrate the BBB to the greatest extent. However, the frequently used parameter for assessing the extent of the CNS distribution is also the ratio of the brain/plasma partition coefficient, Kp,brain. This parameter—calculated for compounds that distribute solely by passive diffusion—is a function of the relative plasma and brain tissue unbound fractions at distribution equilibrium [75]. In our case, most substances, i.e.,arjunic acid, arjunolic acid, arjungenin and sericic acid, have Kp,brain values of less than one, which can result from more extensive binding to proteins in plasma than those in brain tissues. Other explanations can be a significant impairment in the CNS distribution such as the efflux transport at the BBB [75]. However, taking into account the logBB values (Table 2), it can be assumed that these compounds simplyhave a lower CNS-distribution potential contrary to arjunetin and arjunglucoside I: with Kp,brain values of 5.1 and 1.25, respectively. 

There exists the free drug theory that postulates that all the distribution processes of the active substance within biological barriers depend on the unbound drug concentration [70,76]. It must be emphasized that the drug in the blood is present in both the unbound form and bound form to plasma proteins and erythrocytes. In our experiment, two substances, i.e.,arjunetin and arjunglucoside I, have the highest value of the fraction unbound in plasma (0.051 and 0.050, respectively) in contrast to other compounds with Fb values in the range of 0.012 to 0.016. This could confirm earlier suppositions that arjunetin has the greatest ability among the tested compounds to cross the blood–brain barrier. Nevertheless, it is also hypothesized that drugs binding to protein can rapidly dissociate and permeate in vivo through the BBB into the brain tissues [70]. Therefore there may exist some differences between the drug concentration obtained in vivo in the brain and that estimated in vitro based on the free drug concentration. Nevertheless, the ability of most drugs to cross the BBB is nowadays estimated using the free drug fraction theory with reasonably acceptable results [70].

To study the ability of a given substance to cross biological barriers, including the BBB, separation methods are often applied, including high-performance liquid chromatography with the use of systems mimicking the biological environment [77,78]. In our experiment, biomimetic studies were carried out to confirm (or refute) the previous assumptions based on the BBB-pharmacokinetic computational research. For this purpose, micellar liquid chromatography, using non-ionic Brij35(this type of chromatography is called BMC), as well as anionic SDS surfactants, was applied. These methods are commonly used to assess the permeation of a substance through biological barriers [79,80,81,82,83]. The concentration of a surfactant in a micellar mobile phase must be above the critical micellization concentration (cmc), whereas the commonly used stationary phase is octadecyl-modified silica gel [84,85,86]. Due to the wide application of micellar chromatography in the study of the penetration of compounds through biological barriers, it is a recognized technique in biomimetic studies on biologically active compounds.

Since the Brij35 micelle is assumed to be a kind of simple chemical model of the biomembrane, the BMC technique can be useful in describing the biological behaviors of different kinds of organic compounds. It can also mimic many biological processes such as BBB penetration, skin permeability, intestinal absorption and the drug-partitioning process in biological systems [84,85,86], among others. In our research, the logarithms of the retention factor extrapolated to pure water (log km), for both the BMC and SDS systems, have been determined. This parameter is recognized to be an alternative to the logarithm of the n-octanol/water partition coefficient (logPo/w) lipophilicity descriptor.

In the research, each system was previously optimized by selecting the appropriate concentrations of surfactants, selecting the organic modifier and its concentration in the mobile phase. The surfactant solutions were buffered (pH 7.4). Moreover, according to Foley’s equation [26], the interactions performed in the micellar systems have been characterized. Knowledge of the type of interactions between the analyte and the micelle, which in this case is a BBB model, can provide valuable information on the mechanism of interaction between a substance and a barrier. For this purpose, important physicochemical parameters such as *K_MA_*—the analyte–micelle association constant, and P_SW_—the partition coefficient of an analyte between the stationary phase and water, were calculated. Based on the above–mentioned parameters, one can make conclusions about the strength of the analyte interaction with the biological membrane. Such studies can be essential in the context of research on the biological activity of the tested compounds. 

In the previous study [87], it was proved that the logarithm of the analyte–micelle association constant (log*K_MA_*) can directly characterize the passage of substances through the BBB comparable to the logBB pharmacokinetic parameter. Since the Foley’s model describes the retention behaviors of the analyte in the micellar system, which can be treated as a simple BBB model, the parameters contained in it can characterize the biodistribution of the analyte in the BBB area. Very good linear relationships (R^2^> 0.9) between 1/k and C_M_ were obtained for all tested compounds (Figure 1), confirming that Foley’s equation correctly describes the retention of solutes in the tested BMC and SDS chromatographic systems. Log*K_MA_* can be a useful tool for rapid assessment of the ability of a substance to cross the BBB, especially in the early stage of research. The obtained log*K_MA_*-BMC values confirmed that both compounds: arjunetin and arjunglucoside I interact the most with Brij micelles which is recognized as a simply biological membrane model. In the SDS system, the matter is more complicated. Due to the probable electrostatic interactions between the analytes and anionic micelles, as well as the strong retention of compounds, the intercepts for three out of six equations are negative. Unfortunately, the intercepts less than zero donot makephysico-chemical sense because they are equal to the reciprocal of the km parameter, being the retention factor in the system in which the concentration of free surfactant (*C_M_*) in the effluent is equal to zero. However, to eliminate the impact of possible electrostatic interactions, the log (km/*K_MA_*) values calculated from the slopes of Equation (1) were taken into account. These values have been treated as logBB values (logBB-SDS; see Figure 2).

As shown in Figure 2, there are no significant differences between the logBB values obtained using computational and biomimetic methods. The BBB-pharmacokinetic biomimetic studies confirmed that arjunetin and arjunglucoside I can cross the BBB and therefore have the greatest BBB-penetration potential among the tested compounds, while arjunic and arjunolic acids have the smallest one. 

Analyzing the IC_50_ values obtained in the TLC-bioautography assay towards the AChE inhibition, it can be stated that among the tested compounds, arjunolic acid was found to be the strongest inhibitor, whereas arjunetin was the weakest one among the tested compounds. However, the differences in the obtained IC_50_ values are insignificant. Thus, it can be concluded that all compounds have a very similar affinity to AChE which was later confirmed by molecular-docking studies. 

Taking into account the biological potential of other metabolites of plant origin, the compounds tested in the study exhibit a relatively strong inhibitory potential. Previous results on triterpenoids confirmed their AChE-inhibitory potential. In a study on the metabolites from *Centellaasiatica*, asiatic acid was found to be the strongest AChE inhibitor with anIC_50_ value of 15.05 ± 0.05 µM [88]. Also, the metabolites of *Garcinia hombroniana* delivered information on a high inhibitory potential of 2β-hydroxy-3α-O-caffeoyltaraxar-14-en28-oic acid present in the plant [89]. In comparison to Amaryllidaceae alkaloids, like galantamine that is registered as a first-line drug in the treatment of AD and was characterized by anIC_50_ value of 3.520 µM [90], the tested compounds seem to be promising.

As the ARG exhibits the lowest IC_50_ value [mM] among the tested compounds, it was applied in the AChE-inhibition kinetic studies using the colorimetric Ellman’s test [24]. The Michaelis–Menten constant (Km) calculated based on the course of the curve (Figure 6) was found to be 0.000011 mol/L. The obtained relationships show that the rate of substrate–enzyme binding is concentration-dependent and reaches a maximum velocity equal to 2.2 × 10^−5^.

The results of the docking study have been analyzed with respect to the mechanistic interaction pattern that may be significant in the context of interpreting the obtained binding energies and recognizing the role of pharmacophore fragments. The summary given below relies on analyzing the ligand–protein contacts that take place if the distance between any corresponding atom pair is smaller than the arbitrarily accepted value of 0.4 nm. Figure 3A shows the superposition of all of the most favorable ligand, poses whereas Figure 3B shows the most essential ligand–protein interactions. The extremely close match between the superposed structures of all compounds agrees with the previous statement claiming that binding strength is determined by the pharmacophore-like common molecular fragment of all compounds. 

The detailed pattern of ligand–enzyme interactions is illustrated in Figure 3B, from the example of arjunglucoside I (i.e., the compound displaying the lowest IC_50_ value [mM]). However, due to similar orientations of all ligands in the binding cavity, the majority of conclusions can be transferred to the remaining compounds. All ligands prefer roughly the same binding position in the enzyme cavity which enables them to block the catalytic site (the proximity to the catalytic histidine, His440, can be observed). The central fragment of the ligand molecule (composed of aliphatic cyclic moieties) interacts with the aromatic cluster of sidechains, created by His440, Phe290, Trp84, Trp279, Phe288, Phe331, Tyr121, Tyr70 and Tyr334. Such contacts have a character of the CH-π interactions, supported (in some cases, e.g., His44) by hydrogen bonding with the neighboring fragments of ligand. The hydroxyl groups located at the edge of the aliphatic condensed fragment of the ligand interact with Arg289 and Ser286. Both of these contacts occur via hydrogen bonding and, surprisingly, involve backbone fragments of the protein (the ligand can only be a hydrogen bonding donor). One can speculate about an analogous interaction in the case of Ile286 (also a backbone fragment) but, due to the lack of rotation around the peptide bonds in the docking procedure, this was not explicitly observed. Interestingly, the ligand contacts with non-aromatic hydrophobic sidechains are marginal and include only Ile287 and Leu127. Even in these cases, such proximities are rather an opportunistic consequence of much stronger interactions occurring with other adjacent amino-acid residues.

The moiety of type and character varying between molecules (topologically equivalent to the glucopyranose residue in the case of arjunglucoside I, illustrated in Figure 3) is located close to a set of polar amino-acid residues, including Asn85, Ser122, Gln69 and Asp72. The dominating character of the involved interactions is hydrogen bonding, where the considered fragment of the ligand molecule can play the role of both donor and acceptor. In spite of the presence of tryptophan and tyrosine sidechains in close proximity to the glucopyranosidic moiety, no CH-π stacking characteristic for carbohydrate–protein binding was observed. This may explain why this fragment of ligand molecule (or its lack) is not particularly crucial for binding strength; the hydrogen bond donors and acceptors present in this region of cavity can be equally well saturated by water molecules, providing roughly the same balance of energy.

Taking into account the toxicity predicted values, it can be stated that ARG has a low potential for chronic and acute toxicity on fish, Daphnia and green algae.

## 5. Conclusions

The results of the presented studies showed that naturally occurring oleanane-type triterpenes and their glycosides may by active against the AChE enzyme. Therefore, these compounds, especially arjunetin and arjunglucoside I, can be novel drug candidates in the treatment of neurodegenerative diseases with memory impairment including AD and can be an interesting source of further deeper research in this aspect. 

## Figures and Tables

**Table 1:** The chemical structures of the tested compounds.

No.	Name	Chemical Structure
1	Arjunic acid	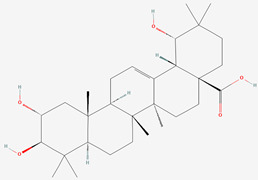
2	Arjunolic acid	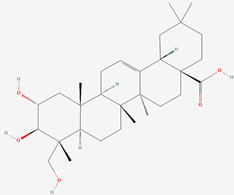
3	Arjungenin	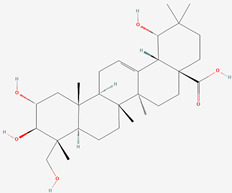
4	Arjunglucoside I	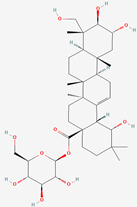
5	Sericic acid	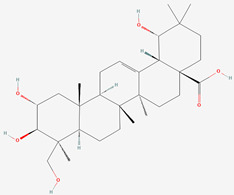
6	Arjunetin	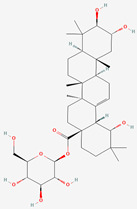

**Table 2:** The BBB-pharmacokinetic and distribution parameters of the analyzed compounds calculated in silico(ACD/Percepta software).

Name	logBB	logPS	logPS,_Fu,brain_	Fu	Fb
Arjunic acid	−0.14	−3.2	−4.9	0.012	0.02
Arjunolic acid	−0.13	−3.2	−5.0	0.012	0.02
Arjungenin	−0.15	−2.9	−4.6	0.016	0.02
Arjunglucoside I	0.12	−2.9	−4.4	0.050	0.04
Sericic acid	−0.15	−2.9	−4.6	0.016	0.02
Arjunetin	0.73	−2.3	−4.3	0.051	0.01

**Table 3:** The IC_50_ values calculated in the TLC-bioautography assay towards the AChE inhibition for the tested standards.

Name	IC_50_ [mg/mL]	IC_50_ [mM]
arjunic acid	10.12	0.0207
arjunolic acid	7.92	0.0162
arjungenin	9.83	0.0195
arjunglucoside I	8.78	0.0132
sericic acid	8.3	0.0164
arjunetin	12.23	0.0188

**Table 4:** Acute and chronic toxicity predicted in silico.

Organism	Duration	Endpoint	Predicted Value [mg/L]
Acute effects			
Fish	96 h	LC50	566.412
Daphnia	48 h		1364.175
Green algae	96 h	EC50	721.889
Chronic effects			
Fish		ChV	56.863
Daphnia			1385.105
Green algae			118.412

## Data Availability

The data presented in this study are available in this article.

## References

[1] (2017). The WHO Brochure “Global Action Plan on the Public Health Response to Dementia 2017–2025”. https://www.who.int/publications/i/item/global-action-plan-on-the-public-health-response-to-dementia-2017-2025.

[2] Wiendl H., Meuth S.G. (2015). Pharmacological Approaches to Delaying Disability Progression in Patients with Multiple Sclerosis. Drugs.

[3] Zuin M., Cherubini A., Volpato S., Ferrucci L., Zuliani G. (2022). Acetyl-cholinesterase-inhibitors slow cognitive decline and decrease overall mortality in older patients with dementia. Sci. Rep..

[4] Smyrska-Wieleba N., Mroczek T. (2023). Natural Inhibitors of Cholinesterases: Chemistry, Structure–Activity and Methods of Their Analysis. Int. J. Mol. Sci..

[5] Santos T.C.D., Gomes T.M., Pinto B.A.S., Camara A.L., Paes A.M.D.A. (2018). Naturally Occurring Acetylcholinesterase Inhibitors and Their Potential Use for Alzheimer’s Disease Therapy. Front. Pharmacol..

[6] Berkov S., Atanasova M., Georgiev B., Bastida J., Doytchinova I. (2022). The Amaryllidaceae alkaloids: An untapped source of acetylcholinesterase inhibitors. Phytochem. Rev..

[7] Atanasov A.G., Zotchev S.B., Dirsch V.M., Orhan I.E., Banach M., Rollinger J.M., Barreca D., Weckwerth W., Bauer R., Bayer E.A. (2021). Natural products in drug discovery: Advances and opportunities. Nat. Rev. Drug Discov..

[8] Bora K.S., Sharma A. (2011). Phytochemical and pharmacological potential of *Medicago sativa*: A review. Pharm. Biol..

[9] Liu X.-G., Sun Y.-Q., Bian J., Han T., Yue D.-D., Li D.-Q., Gao P.-Y. (2019). Neuroprotective effects of triterpenoid saponins from *Medicago sativa* L. against H2O2-induced oxidative stress in SH-SY5Y cells. Bioorg. Chem..

[10] Li Z., Liu Y., Wang L., Liu X., Chang Q., Guo Z., Liao Y., Pan R., Fan T.P. (2014). Memory-Enhancing Effects of the Crude Extract of *Polygala tenuifolia* on Aged Mice. Evid.-Based Complement. Altern. Med..

[11] Li C., Yang J., Yu S., Chen N., Xue W., Hu J., Zhang D. (2008). Triterpenoid Saponins with Neuroprotective Effects from the Roots of *Polygala tenuifolia*. Planta Med..

[12] Son I.H., Park Y.H., Lee S.I., Yang H.D., Moon H.-I. (2007). Neuroprotective Activity of Triterpenoid Saponins from Platycodi radix Against Glutamate-induced Toxicity in Primary Cultured Rat Cortical Cells. Molecules.

[13] Yaidikar L., Thakur S. (2015). Arjunolic acid, a pentacyclictriterpenoidalsaponin of *Terminalia arjuna* bark protects neurons from oxidative stress associated damage in focal cerebral ischemia and reperfusion. Pharmacol. Rep..

[14] Toropov A.A., Toropova A.P., Beeg M., Gobbi M., Salmona M. (2017). QSAR model for blood-brain barrier permeation. J. Pharmacol. Toxicol. Methods.

[15] Van Bree J.B.M.M., De Boer A.G., Danhof M., Breimer D.D. (1992). Drug transport across the blood-brain barrier. Pharm. Weekbl..

[16] Iyer M., Mishra R., Han Y., Hopfinger A.J. (2002). Blood–Brain Barrier Partitioning of Organic Molecules Using Membrane-Interaction QSAR Analysis. Pharm. Res..

[17] Ekins S., Waller C.L., Swaan P.W., Cruciani G., Wrighton S.A., Wikel J.H. (2000). Progress in predicting human ADME parameters *in silico*. J. Pharmacol. Toxicol. Methods.

[18] Anastas P., Eghbali N. (2010). Green chemistry: Principles and practice. Chem. Soc. Rev..

[19] Kukula-Koch W., Mroczek T. (2015). Application of hydrostatic CCC-TLC-HPLC-ESI-TOF-MS for the bioguided fractionation of anticholinesterase alkaloids from *Argemonemexicana* L. roots. Anal. Bioanal. Chem..

[20] Hanwell M.D., Curtis D.E., Lonie D.C., Vandermeersch T., Zurek E., Hutchison G.R. (2012). Avogadro: An advanced semantic chemical editor, visualization, and analysis platform. J. Cheminform..

[21] Rappe A.K., Casewit C.J., Colwell K.S., Goddard W.A., Skiff W.M. (1992). UFF, a full periodic table force field for molecular mechanics and molecular dynamics simulations. J. Am. Chem. Soc..

[22] Trott O., Olson A.J. (2010). AutoDockVina:Improving the speed and accuracy of docking with a new scoring function, efficient optimization, and multithreading. J. Comput. Chem..

[23] Tarabasz D., Szczeblewski P., Laskowski T., Płaziński W., Baranowska-Wójcik E., Szwajgier D., Kukula-Koch W., Meissner H.O. (2022). The Distribution of Glucosinolates in Different Phenotypes of Lepidiumperuvianum and Their Role as Acetyl- and Butyrylcholinesterase Inhibitors-In Silico and In Vitro Studies. Int. J. Mol. Sci..

[24] Ellman G.L., Lourtney D.K., Andres V., Gmelin G. (1961). A new and rapid colorimetric determination of acetylcholinesterase activity. Biochem. Pharmacol..

[25] Szwajgier D., Baranowska-Wójcik E. (2019). Terpenes and Phenylpropanoids as Acetyl- and Butyrylcholinesterase Inhibitors: A Comparative Study. Curr. Alzheimer Res..

[26] Foley J.P. (1990). Critical compilation of solute-micelle binding constants and related parameters from micellar liquid chromatographic measurements. Anal. Chim. Acta.

[27] Janicka M., Śliwińska A., Sztanke M., Sztanke K. (2022). Combined Micellar Liquid Chromatography Technique and QSARs Modeling in Predicting the Blood–Brain Barrier Permeation of Heterocyclic Drug-like Compounds. Int. J. Mol. Sci..

[28] Birks J., Evans J.G. (2009). *Ginkgo biloba* for cognitive impairment and dementia. Cochrane Database Syst. Rev..

[29] Joo S.S., Lee D.I. (2005). Potential effects of microglial activation induced by ginsenoside Rg3 in rat primary culture: Enhancement of type a macrophage scavenger receptor expression. Arch. Pharm. Res..

[30] Chen F., Eckman E.A., Eckman C.B. (2006). Reductions in levels of the Alzheimer’s amyloid β peptide after oral administration of ginsenosides. FASEB J..

[31] Li N., Liu B., Dluzen D.E., Jin Y. (2007). Protective effects of ginsenoside Rg2 against glutamateinduced neurotoxicity in PC12 cells. J. Ethnopharmacol..

[32] Joo S.S., Yoo Y.M., Ahn B.W., Nam S.Y., Kim Y.B., Hwang K.W., Lee D.I. (2008). Prevention of inflammation-mediated neurotoxicity by Rg3 and its role in microglial activation. Biol. Pharm. Bull..

[33] Shieh P.C., Tsao C.W., Li J.S., Wu H.T., Wen Y.J., Kou D.H., Cheng J.T. (2008). Role of pituitary adenylate cyclase-activating polypeptide (PACAP) in the action of ginsenoside Rh2 against betaamyloid-induced inhibition of rat brain astrocytes. Neurosci. Lett..

[34] Wakabayashi I., Yasui K. (2000). Wogonin inhibits inducible prostaglandin E2 production in macrophages. Eur. J. Pharmacol..

[35] Park B.K., Heo M.Y., Park H., Kim H.P. (2001). Inhibition of TPA-induced cyclooxygenase-2 expression and skin inflammation in mice by wogonin, a plant flavone from *Scutellaria radix*. Eur. J. Pharmacol..

[36] Nakamura N., Hayasaka S., Zhang X.Y., Nagaki Y., Matsumoto M., Hayasaka Y., Terasawa K. (2003). Effects of baicalein, baicalin, and wogonin on interleukin-6 and interleukin-8 expression, and nuclear factor-kb binding activities induced by interleukin-1beta in human retinal pigment epithelial cell line. Exp. Eye Res..

[37] Suk K., Lee H., Kang S.S., Cho G.J., Choi W.S. (2003). Flavonoid baicalein attenuates activationinduced cell death of brain microglia. J. Pharmacol. Exp. Ther..

[38] La Vecchia C., Bosetti C. (2007). Diet and cancer risk in Mediterranean countries. Hungar. Med. J..

[39] Trichopoulou A., Lagiou P., Kuper H., Trichopoulos D. (2000). Cancer and Mediterranean dietary traditions. Cancer Epidemiol. Biomark. Prev..

[40] Visioli F., Grande S., Bogani P., Galli C. (2004). The role of antioxidants in the Mediterranean diets: Focus on cancer. Eur. J. Cancer Prev..

[41] Ninfali P., Mea G., Giorgini S., Rocchi M., Bacchiocca M. (2005). Antioxidant capacity of vegetables, spices, and dressings relevant to nutrition. Br. J. Nutr..

[42] Santos-Neto L.L.D., de Vilhena Toledo M.A., Medeiros-Souza P., de Souza G.A. (2006). The use of herbal medicine in Alzheimer’s disease--A systematic review. Evid.-Based Complement. Altern. Med..

[43] Bozin B., Mika-Dukic N., Samojlik I., Jovin E. (2007). Antimicrobial and antioxidant properties of rosemary and sage (*Rosmarinus officinalis* L. and *Salvia officinalis* L., *Lamiaceae*) essential oils. J. Agric. Food. Chem..

[44] Cheung S., Tai J. (2007). Anti-proliferative and antioxidant properties of rosemary *Rosmarinus officinalis*. Oncol. Rep..

[45] Imanshahidi M., Hosseinzadeh H. (2006). The pharmacological effects of Salvia species on the central nervous system. Phytother. Res..

[46] Bhat J.U., Nizami Q., Asiaf A., Parray S.A., Ahmad S.T., Aslam M., Khanam R., Mujeeb M., Umar S., Siddiqi A. (2012). Anticonvulsant activity of methanolic and aqueous extracts of Melissa parviflora in experimentally induced Swiss albino mice. EXCLI J..

[47] Hosseinzadeh H., Ramezani M., Shafaei H., Taghiabadi E. (2013). Anticonvulsant effect of Berberisintegerrima L. root extracts in mice. J. Acupunct. Meridian Stud..

[48] Ya’u J., Yaro A.H., Malami S., Musa M.A., Abubakar A., Yahaya S.M., Chindo B.A., Anuka J.A., Hussaini I.M. (2015). Anticonvulsant activity of aqueous fraction of *Carissa edulis* root bark. Pharm. Biol..

[49] Sankari M., Chitra V., Silambujanaki P., Raju D. (2010). Anticonvulsant activity of ethanolic extract of *Aegle marmelos* (leaves) in mice. Int. J. Pharmtech Res..

[50] Anaka O.N., Ozolua R.I., Ikpefan E.O., Ogieva D. (2014). Anticonvulsant activity of the aqueous extract of *Allium cepa* L. (Amaryllidaceae) in rats and mice. J. Pharm. Biores..

[51] Showraki A., Emamghoreishi M., Oftadegan S. (2016). Anticonvulsant Effect of the Aqueous Extract and Essential Oil of *CarumCarvi* L. Seeds in a Pentylenetetrazol Model of Seizure in Mice. Iran. J. Med. Sci..

[52] Amabeoku G.J., Oluchi N.M., Davids T., Fakude S., Gqwaka A., Mbai F., Pieterse R., Shaik A. (2014). Evaluation of the anticonvulsant activity of the leaf methanol extract of *Crassula arborescens* (Mill) Willd. (Crassulaceae) in mice. J. Pharm. Pharmacol..

[53] Chaulya N.C., Haldar P.K., Mukherjee A. (2011). Antidiabetic activity of methanol extract of rhizomes of *CyperustegetumRoxb* (Cyperaceae). Acta Pol. Pharm..

[54] Chinchawade A.B., Deshmukh D.B., Gaikwad D.D., Grampurohit N.D. (2013). Anticonvulsant Activity of Chloroform Extract of Bark and Root of *Erythrinavariegata* L.. Int. J. Pharm. Clin. Res..

[55] Nishanthi N., Mohanambal E., Devdass G., Saravanan D., Narendiran S., Vijayakumar B. (2012). Anticonvulsant Activity of *Peperomiatetraphylla* (G. Forst., Hook. &Arn.). Int. J. Nov. Trends Pharm. Sci..

[56] Barua C.C., Begum S.A., Barua A.G., Borah R.S., Lahkar M. (2013). Anxiolytic and anticonvulsant activity of methanol extract of leaves of *Alternanthera brasiliana* (L.) Kuntze (Amaranthaceae) in laboratory animals. Indian J. Exp. Biol..

[57] Khan I., Karim N., Ahmad W., Abdelhalim A., Chebib M. (2016). GABA-A Receptor Modulation and Anticonvulsant, Anxiolytic, and Antidepressant Activities of Constituents from *Artemisia indica* Linn. Evid.-Based Complement. Altern. Med..

[58] Li D.-Q., Zhou L., Wang D., Wu J., Li L.Z., Huang X.X., Liu Q.B., Wu Y.Y., Lin S., Yang J.Y. (2016). Neuroprotective oleanane triterpenes from the roots of *Bupleurumchinense*. Bioorg. Med. Chem. Lett..

[59] Gao Y., Yan H., Jin R., Lei P. (2016). Antiepileptic activity of total triterpenes isolated from Poriacocos is mediated by suppression of aspartic and glutamic acids in the brain. Pharm. Biol..

[60] Srivastava G., Garg A., Misra R.C., Chanotiya C.S., Ghosh S. (2020). Transcriptome analysis and functional characterization of oxidosqualenecyclases of the arjuna triterpene saponin pathway. Plant Sci..

[61] Mohanty I.R., Borde M., Kumar S., Maheshwari U. (2019). Dipeptidyl peptidase IV Inhibitory activity of Terminalia arjuna attributes to its cardioprotective effects in experimental diabetes: *In silico*, *in vitro* and *in vivo* analyses. Phytomedicine.

[62] Pawar R.S., Bhutani K.K. (2005). Effect of oleanane triterpenoids from Terminalia arjuna—A cardioprotective drug on the process of respiratory oxyburst. Phytomedicine.

[63] Kapoor D., Vijayvergiya R., Dhawan V. (2014). Terminalia arjuna in coronary artery disease: Ethnopharmacology, pre-clinical, clinical & safety evaluation. J. Ethnopharmacol..

[64] Pugazhendhi A., Shafreen R.B., Devi K.P., Suganthy N. (2018). Assessment of antioxidant, anticholinesterase and antiamyloidogenic effect of Terminalia chebula, Terminalia arjuna and its bioactive constituent 7-Methyl gallic acid—An in vitro and in silico studies. J. Mol. Liq..

[65] Gupta D., Kumar M. (2016). Evaluation of in vitro antimicrobial potential and GC-MS analysis of *Camellia sinensis* and *Terminalia arjuna*. Biotechnol. Rep..

[66] Mandal S., Patra A., Samanta A., Roy S., Mandal A., Mahapatra T.D., Pradhan S., Das K., Nandi D.K. (2013). Analysis of phytochemical profile of *Terminaliaarjuna* bark extract with antioxidative and antimicrobial properties. Asian Pac. J. Trop. Biomed..

[67] Dube N., Nimgulkar C., Bharatraj D.K. (2017). Validation of therapeutic anti-inflammatory potential of ArjunaKsheeraPaka—A traditional Ayurvedic formulation of *Terminalia arjuna*. J. Tradit. Complement. Med..

[68] Ahmad M.S., Ahmad S., Gautam B., Arshad M., Afzal M. (2014). *Terminalia arjuna*, a herbal remedy against environmental carcinogenicity: An in vitro and in vivo study. Egypt. J. Med. Hum. Genet..

[69] Bhattacharjee B., Pal P.K., Ghosh A.K., Mishra S., Chattopadhyay A., Bandyopadhyay D. (2019). Aqueous bark extract of *Terminalia arjuna* protects against cadmium-induced hepatic and cardiac injuries in male Wistar rats through antioxidative mechanisms. Food Chem. Toxicol..

[70] Tanaka H., Mizojiri K. (1999). Drug-protein binding and blood-brain barrier permeability. J. Pharmacol. Exp. Ther..

[71] Platts J.A., Abraham M.H., Zhao Y.H., Hersey A., Ijaz L., Butina D. (2001). Correlation and prediction of a large blood-brain distribution data set--an LFER study. Eur. J. Med. Chem..

[72] Young R.C., Mitchell R.C., Brown T.H., Ganellin C.R., Griffiths R., Jones M., Rana K.K., Saunders D., Smith I.R., Sore N.E. (1988). Development of a new physicochemical model for brain penetration and its application to the design of centrallyacting H2 receptor histamine antagonists. J. Med. Chem..

[73] Fantini S., Sassaroli A., Tgavalekos K.T., Kornbluth J. (2016). Cerebral blood flow and autoregulation: Current measurement techniques and prospects for noninvasive optical methods. Neurophotonics.

[74] Liu X., Smith B.J., Chen C., Callegari E., Becker S.L., Chen X., Cianfrogna J., Doran A.C., Doran S.D., Gibbs J.P. (2005). Use of a Physiologically Based Pharmacokinetic Model to Study the Time to Reach Brain Equilibrium: An Experimental Analysis of the Role of Blood-Brain Barrier Permeability, Plasma Protein Binding, and Brain Tissue Binding. J. Pharmacol. Exp. Ther..

[75] Kalvass J.C., Maurer T.S., Pollack G.M. (2007). Use of plasma and brain unbound fractions to assess the extent of brain distribution of 34 drugs: Comparison of unbound concentration ratios to in vivo p-glycoprotein efflux ratios. Drug Metab. Dispos..

[76] Hammarlund-Udenaes M., Fridén M., Syvänen S., Gupta A. (2008). On the rate and extent of drug delivery to the brain. Pharm. Res..

[77] Ciura K., Kapica H., Dziomba S., Kawczak P., Belka M., Bączek T. (2020). Biopartitioning micellar electrokinetic chromatography—Concept study of cationic analytes. Microchem. J..

[78] Ciura K., Dziomba S. (2020). Application of separation methods for in vitro prediction of blood–brain barrier permeability—The state of the art. J. Pharm. Biomed. Anal..

[79] Escuder-Gilabert L., Molero-Monfort M., Villanueva-Camañas R.M., Sagrado S., Medina-Hernández M.J. (2004). Potential of biopartitioning micellar chromatography as an in vitro technique for predicting drug penetration across the blood–brain barrier. J. Chromatogr. B.

[80] Martínez-Pla J.J., Martín-Biosca Y., Sagrado S., Villanueva-Camañas R.M., Medina-Hernández M.J. (2004). Evaluation of the pH effect of formulations on the skin permeability of drugs by biopartitioning micellar chromatography. J. Chromatogr. A.

[81] Hadjmohammadi M., Salary M. (2013). Biopartitioning micellar chromatography with sodium dodecyl sulfate as a pseudo α1-acid glycoprotein to the prediction of protein—Drug binding. J. Chromatogr. B.

[82] Tsopelas F., Danias P., Pappa A., Tsantili-Kakoulidou A. (2020). Biopartitioning micellar chromatography under different conditions: Insight into the retention mechanism and the potential to model biological processes. J. Chromatogr. A.

[83] Dobričić V., Nikolic K., Vladimirov S., Čudina O. (2014). Biopartitioning micellar chromatography as a predictive tool for skin and corneal permeability of newly synthesized 17β-carboxamide steroids. Eur. J. Pharm. Sci..

[84] Molero-Monfort M., Escuder-Gilabert L., Villanueva-Camanas R.M., Sagrado S., Medina-Hernández M.J. (2001). Biopartitioning micellar chromatography: An in vitro technique for predicting human drug absorption. J. Chromatogr. B.

[85] Escuder-Gilabert L., Martinez-Pla J.J., Sagrado S., Villanueva-Camañas R.M., Medina-Hernández M.J. (2003). Biopartitioning micellar separation methods: Modelling drug absorption. J. Chromatogr. B.

[86] Escuder-Gilabert L., Sanchis-Mallols J.M., Sagrado S., Medina-Hernández M.J., Villanueva-Camañas R.M. (1998). Chromatographic quantitation of the hydrophobicity of ionic compounds by the use of micellar mobile phases. J. Chromatogr. A..

[87] Stępnik K. (2021). Biomimetic Chromatographic Studies Combined with the Computational Approach to Investigate the Ability of Triterpenoid Saponins of Plant Origin to Cross the Blood-Brain Barrier. Int. J. Mol. Sci..

[88] Jusril N.A., Muhamad Juhari A.N.N., Abu Bakar S.I., MdSaad W.M., Adenan M.I. (2020). Combining In Silico and In Vitro Studies to Evaluate the Acetylcholinesterase Inhibitory Profile of Different Accessions and the Biomarker Triterpenes of *Centellaasiatica*. Molecules.

[89] Jamila N., Khairuddean M., Yeong K.K., Osman H., Murugaiyah V. (2014). Cholinesterase inhibitory triterpenoids from the bark of *Garcinia hombroniana*. J. Enzym. Inhib. Med. Chem..

[90] Stavrakov G., Philipova I., Lukarski A., Atanasova M., Zheleva D., Zhivkova Z.D., Ivanov S., Atanasova T., Konstantinov S., Doytchinova I. (2020). Galantamine-curcumin hybrids as dual-site binding acetylcholinesterase inhibitors. Molecules.

